# A Comparative Study of Iron Status in Subclinical Hypothyroid and Euthyroid Subjects in a Tertiary Care Hospital

**DOI:** 10.7759/cureus.52007

**Published:** 2024-01-10

**Authors:** Thokati G Swapnika, S S Sabitha Rani, Satish Dipankar, Afreen Begum H Itagi, Immadi S Vamshidhar

**Affiliations:** 1 Biochemistry, Krishna Institute of Medical Sciences (Hospital Group), Hyderabad, IND; 2 Pathology and Laboratory Medicine, Government Medical College Bhadradri Kothagudem, Old Paloncha, IND; 3 Physiology, All India Institute of Medical Sciences, Mangalagiri, IND

**Keywords:** iron deficiency anemia (ida), hemoglobin (hb), thyrotropin (tsh), subclinical hypothyroidism, anemia

## Abstract

Background: The physiological equilibrium of the entire human body's metabolism is significantly influenced by thyroid hormones. Subclinical hypothyroidism, which is often concealed, is connected to iron deficiency anemia and various other hematological disorders. We in the current study tried to determine the prevalence and severity of iron deficiency anemia and investigate the correlation of subclinical hypothyroidism with iron deficiency.

Methods: A total of 100 subjects included in the study were divided into two groups. Group 1 included 50 cases with subclinical hypothyroidism, and Group 2 included 50 healthy age- and sex-matched controls. Hemoglobin (Hb) levels were measured within 24 hours of sample collection using a Sysmex automated cell counter (Kobe, Hyogo, Japan). Thyroid hormones (free triiodothyronine (fT3), free thyroxine (fT4), and thyroid-stimulating hormone (TSH)) were measured.

Results: Out of 50 cases, 48 (96%) have iron deficiency anemia, and 52 (104%) have subclinical hypothyroidism. Among the cases with iron deficiency anemia, 43 (86%) also have subclinical hypothyroidism. There was a negative correlation between thyrotropin (TSH) and Hb levels. Pearson's correlation coefficient "r" values were -0.86408. The serum ferritin levels of cases were decreased as compared to the healthy controls, and the difference in means for cases and controls in terms of serum ferritin is also statistically significant.

Conclusion: The prevalence of anemia in subclinical hypothyroidism is significantly elevated, and considering the absence of significant clinical manifestations in the early stages, it is recommended to routinely conduct investigations for early detection, facilitating prompt management. Consequently, our study emphasizes that both overt and subclinical hypothyroidism should be recognized as risk factors for the development of iron deficiency anemia.

## Introduction

Thyroid diseases are highly prevalent globally, with India being no exception [1]. The burden of thyroid disorders in the general population is substantial [2]. About 50% of people in the community have microscopic thyroid nodules, 5% of women experience overt hypothyroidism or hyperthyroidism, 15% have palpable goiters, 3.5% have occult papillary carcinoma, and 10% exhibit abnormal thyroid-stimulating hormone (TSH) levels [3,4]. Projections suggest that around 42 million people in India are affected by thyroid diseases [5]. Hypothyroidism, a common thyroid disorder, has been the subject of a recent study in India. A population-based study in Cochin involving 971 adult subjects reported a prevalence of hypothyroidism at 3.9% [6]. This condition was more common in women (11.4%) than in men (6.2%), and its prevalence increased with age. Subclinical hypothyroidism is characterized by elevated TSH levels in the presence of normal thyroxine (T4) concentrations [7]. Hypothyroidism can lead to various forms of anemia. These anemias can be microcytic, macrocytic, or normocytic [8]. Microcytic anemia in hypothyroidism is often due to iron deficiency, resulting from malabsorption associated with hypothyroidism and menorrhagia due to hormonal imbalances. Patients with hypothyroidism frequently exhibit low ferritin levels. Iron is a critical component in several enzymes, including thyroid peroxidase (TPO), involved in thyroid hormone biosynthesis [9]. Iron deficiency can significantly reduce T4 and triiodothyronine (T3) levels and inhibit the conversion of T4 to T3 [10].

Normal levels of thyroid hormones play a role in hemoglobin (Hb) synthesis in adults, and low levels of thyroid hormones affect the hematopoietic process, leading to anemia by slowing oxygenation. Studies have shown that nutritional iron deficiency can reduce T4 and T3 levels and hamper the conversion of T4 to T3 [7,10-12]. Additionally, thyroid hormones are thought to influence hematopoiesis by increasing erythropoietin production or hematopoietic factors in non-erythroid cells. In overt hypothyroidism, anemia is a common finding, and in up to 25% of patients with hypothyroidism, anemia normalizes with T4 replacement, even in the presence of normal iron, vitamin B12, and folate levels. Few studies have examined the link between subclinical hypothyroidism and anemia, and anemia is not typically listed as a consequence of untreated subclinical hypothyroidism [12]. Due to a lack of large cohort studies, a definitive association between subclinical hypothyroidism and iron deficiency anemia is yet to be established, posing management challenges. One study found that women with subclinical hypothyroidism often exhibited sideropenia and recommended routine ferritin level checks for these patients. However, another study reported no change in Hb or hematocrit levels upon the restoration of euthyroidism in women with subclinical hypothyroidism [7]. Serum ferritin, a key iron storage compound, is primarily found in reticuloendothelial cells and can be influenced by thyroid function. Iron deficiency can impair the body's ability to produce its thyroid hormone, potentially increasing the need for thyroid medication [13]. This suggests that serum ferritin measurements could be valuable in assessing thyroid hormone action in peripheral tissues [14]. The present study was conducted to explore subclinical hypothyroid patients, determine the prevalence and severity of iron deficiency anemia in this group, and investigate the effects of subclinical hypothyroidism on body iron stores.

## Materials and methods

This cross-sectional study was conducted in the Department of Biochemistry, Osmania Medical College, Hyderabad, India. Ethical clearance was obtained to conduct this study, from the said institution's Institutional Ethics Committee (approval number: ECR/300/Inst/AP/2013/RR-16). Informed consent was taken from all the individuals who took part in the study.

Sampling size calculation

The formula used to calculate sample size was N=4PQ/d2=4x9x91/36=91. The calculated sample size was 91, and we have taken 100 subjects where P is the prevalence rate which was taken as 9% (from previous studies) [15], Q is 100-P, and d is the margin of error kept at 6.

A total of 100 human subjects included in the study were divided into two groups. Group 1 included 50 cases with subclinical hypothyroidism, and Group 2 included 50 healthy age- and sex-matched controls. The threshold value of TSH was 4.0 mIU/L; those subjects with values of TSH greater than 4.0 mIU/L were taken as subclinical hypothyroidism, and those with TSH values lower than 4.0 were taken as normal cases. The inclusion and exclusion criteria for the present study are shown below in Table 1 and Table 2, respectively.

**Table 1:** Inclusion criteria

Inclusion criteria	
Group 1	Patients with subclinical hypothyroid in the age group of 14-55 years.
Group 2	Healthy volunteers in the age group of 14-55 years without any exclusion conditions who have come for routine screening of thyroid profile.

**Table 2:** Exclusion criteria

Exclusion criteria	
1	Patients with established thyroid disease and who are on treatment
2	Surgical thyroidectomy and replacement therapy
3	Pregnant women and lactating mothers
4	Hemolytic anemia
5	Patients under treatment that might affect blood parameters such as steroids
6	Autoimmune disorders other than hypothyroidism

Demographic information, including age, sex, height, and weight, was recorded for all the participants. A 5 ml blood sample was obtained from each subject, with 3 ml placed in a serum tube and 2 ml in K2 ethylenediaminetetraacetic acid (EDTA) tubes. The serum tube was centrifuged after allowing for adequate clotting, and the separated serum was divided into aliquots for subsequent thyroid profile, ferritin, and soluble transferrin receptor level testing. Plasma was separated from the EDTA tubes and used for a complete blood count (CBC) analysis. Additionally, a drop of blood was collected for peripheral blood smear analysis using a needle prick. The aliquoted samples were stored at -20°C for future use. Hb levels were measured within 24 hours of sample collection using a Sysmex automated cell counter (Kobe, Hyogo, Japan). Thyroid hormones (free triiodothyronine (fT3), free thyroxine (fT4), and TSH) were measured by electrochemiluminescence immunoassay (ECLIA) competitive assay in Cobas 6000 automated hormone analyzer (Roche Diagnostics, Indianapolis, Indiana, United States). Serum iron, ferritin, and transferrin levels were also measured by the immunoassay method using a Cobas 6000 automated analyzer.

Anemia was characterized by Hb levels below 12 g/dL in women and 13 g/dL in men [16]. Iron deficiency anemia was identified by serum iron levels <60 μg/dL, iron-binding capacity exceeding 215 μg/dL, ferritin levels <10 ng/dL, and the presence of microcytosis and hypochromia in the peripheral blood smear.

Statistical analysis

Data were analyzed using IBM SPSS Statistics for Windows, Version 21.0 (Released 2012; IBM Corp., Armonk, New York, United States). The collected data were tabulated in an Excel spreadsheet (Microsoft Corporation, Redmond, Washington, United States) and subsequently analyzed. Continuous variables are presented as mean±standard deviation (SD), while categorical variables are expressed as frequencies and percentages. Categorical variables were analyzed using the chi-squared test. Pearson's correlation was applied to identify the relationships between variables. A significance level of p≤0.05 was considered for all statistical results.

## Results

The present study was undertaken in the Department of Biochemistry, Osmania Medical College and Osmania General Hospital, Hyderabad, India. A total of 100 participants were recruited for the study which included 50 patients of subclinical hypothyroidism as cases and 50 healthy individuals as controls. Table 3 illustrates the distribution of subjects with subclinical hypothyroidism based on age groups. The prevalence of subclinical hypothyroidism in each age category is presented numerically and as a percentage. The findings reveal that the highest proportion of subjects with subclinical hypothyroidism falls within the 31-40 age group, succeeded by the 21-30 age group, and the mean age was 34.22±4.5 years. Conversely, the 51-60 age group exhibits the smallest percentage of subjects with subclinical hypothyroidism. The mean age of controls was 31.86±3.22 years.

**Table 3:** Distribution of the study subjects with subclinical hypothyroidism according to age group

Age group distribution	Cases	Controls
	Frequency (%)	Frequency (%)
<20	6 (12%)	8 (16%)
21-30	10 (20%)	15 (30%)
31-40	20 (40%)	14 (28%)
41-50	10 (20%)	8 (16%)
51-60	4 (8%)	5 (10%)

In the cases of subclinical hypothyroidism, females were 78% and males were 22%, and in the control subjects, 66% subjects were females and 34% subjects were males. Table 4 compares the biochemical parameters of cases of subclinical hypothyroidism and controls. The parameters include thyroid hormones (TSH, FT4, FT3), Hb, mean corpuscular volume (MCV), serum iron, serum ferritin, and total iron-binding capacity (TIBC). There are significant differences in all of the biochemical parameters between cases of subclinical hypothyroidism and controls. The TSH levels in cases are significantly higher than in controls, and the FT4 and FT3 levels in cases are significantly lower than in controls, confirming the reduced production of thyroid hormones. The Hb levels in cases are significantly lower than in controls, suggesting that subclinical hypothyroidism may be associated with anemia. The MCV levels in cases are not significantly different from controls, indicating that the red blood cells' size remains normal. The serum iron levels in cases are significantly lower than in controls, suggesting that subclinical hypothyroidism may be associated with iron deficiency. The serum ferritin levels in cases are not significantly different from controls, indicating that the ferritin stores in cases are not abnormally low. The TIBC levels in cases are significantly higher than in controls. Overall, the findings in cases of subclinical hypothyroidism are associated with several biochemical abnormalities related to iron metabolism. These abnormalities may contribute to the symptoms and signs of subclinical hypothyroidism, such as fatigue, weight gain, and difficulty concentrating.

**Table 4:** Comparison of biochemical parameters in the cases of subclinical hypothyroidism and controls recorded in the study TSH: thyroid-stimulating hormone; fT4: free thyroxine; fT3: free triiodothyronine; Hb: hemoglobin; MCV: mean corpuscular volume; S. Iron: serum iron; S. Ferritin: serum ferritin; TIBC: total iron-binding capacity; *: significant

Parameter	Cases	Controls	p-value
TSH (mIU/L)	8.17±3.11	2.73±0.96	0.001*
fT4 (ng/dL)	5.16±2.52	12.1±2.95	0.012*
fT3 (pg/ml)	2.06±1.23	6.87±1.5	0.001*
Hb (g/dL)	9.57±2.7	12.89±1.99	0.011*
MCV (fL)	84.4±5.1	87.66±3.5	0.347
S. Iron (ug/dL)	52.2±28.6	108.2±50.09	0.004*
S. Ferritin (ng/ml)	17.2±12.7	113.7±77.8	0.001*
TIBC (ug/dL)	395.48±61.28	340.19±37.81	0.002*

**Figure 1:**
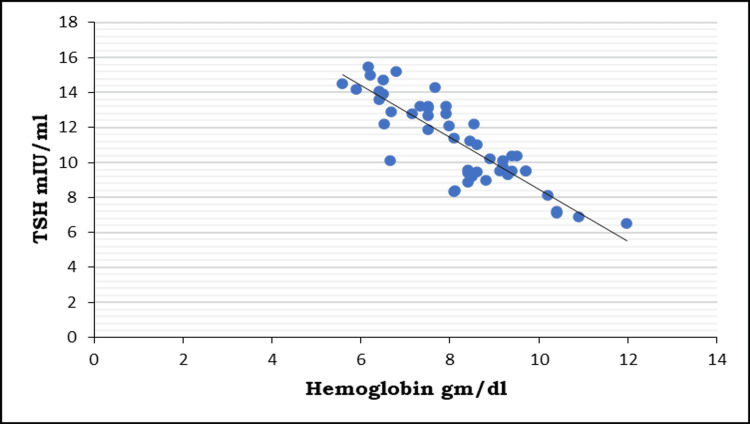
Correlation of TSH with hemoglobin levels in the cases of the study TSH: thyroid-stimulating hormone

Out of 50 cases, 48 have iron deficiency anemia, and 52 have subclinical hypothyroidism. Among the cases with iron deficiency anemia, 43 also have subclinical hypothyroidism (Table 5). Among the cases with subclinical hypothyroidism, seven also have iron deficiency anemia. This may be suggestive of a positive association between iron deficiency anemia and subclinical hypothyroidism. The correlation of TSH with Hb levels in the cases of the study is shown below in Figure 1.

**Table 5:** Comparison of the existence of iron deficiency anemia in cases and controls of the study

Iron deficiency anemia	Subclinical hypothyroidism	
	Present	Absent
Present	43 (86%)	5 (10%)
Absent	7 (14%)	45 (90%)
Total	50 (100%)	50 (100%)

In the correlation between Hb and thyroid hormone statuses within the study population, it was observed that a negative correlation exists between thyrotropin (TSH) and Hb level. Pearson's correlation coefficient "r" values were -0.86408. Consequently, a decrease in Hb levels is associated with an elevation in TSH values. Furthermore, the p-value underscores that these correlations are statistically highly significant.

## Discussion

Subclinical hypothyroidism is widely prevalent, affecting 4-20% of the adult population. It is more frequently observed in women, elderly individuals, and iodine-sufficient populations. The primary cause of subclinical hypothyroidism is chronic autoimmune thyroiditis, specifically linked to anti-TPO antibodies, a condition known as Hashimoto's thyroiditis [17]. People with subclinical hypothyroidism frequently do not display any noticeable symptoms. However, when symptoms manifest, they tend to be nonspecific and may resemble those observed in overt hypothyroidism, such as fatigue, weakness, weight gain, cold intolerance, and constipation. Anemia is a public health problem in India and may be precipitated by conditions such as hypothyroidism [18]. Anemia may precede the onset of other symptoms associated with thyroid diseases. In hypothyroidism, there is a reduction in the development of all myeloid as well as erythroid cell lines, and achieving euthyroid state results in the normalization of all hematological parameters. Normocytic anemia is sometimes linked to a deficiency in thyroid hormones, rather than nutritional deficits. Microcytic anemia is commonly associated with iron malabsorption and menorrhagia. In the context of hypothyroidism, iron malabsorption could be a consequence of achlorhydria [19].

In this study, both cases and controls belonged to the age group of 14-65 years. No significant differences were observed between cases (Group 1) and controls (Group 2) within this age range, indicating that the two groups were comparable over all 8% females with comorbidities and 5% males with comorbidities which included metabolic syndrome, dyslipidemia, and cardiovascular disease. In Group 1 (cases), the TSH mean±SD was 8.17±3.316, while in Group 2 (controls), it was 2.73±0.962. The disparity in means between cases and controls is statistically significant (p=0.001). Additionally, the serum ferritin levels of cases were decreased as compared to the healthy controls, and the difference in means for cases and controls in terms of serum ferritin is also statistically significant (p=0.001). In addition, the serum level of iron was found to decrease, while that of TIBC increased in patients suffering from subclinical hypothyroidism as compared to healthy controls. These results are in accordance with other studies that reported that iron deficiency may be associated with low levels of thyroid hormones [14,19].

In a similar study, Mishra et al. [17] identified that 18.22% of recently diagnosed subclinical hypothyroid patients experienced iron deficiency anemia, with a considerable proportion exhibiting hemoglobin levels below 10 g%. Das et al. [20] reported a 26.6% prevalence of anemia in the subclinical hypothyroid group. Larsson [21] found that 52% (13 out of 25) of patients with hypothyroidism had iron deficiency anemia. Kulkarni and Jadhav [22] in their study observed the existence of microcytic hypochromic morphology in 22.72% of patients with thyroid deficiency. Akhter et al. [23] reported a significant difference in thyroid hormone status in iron-deficient individuals, potentially reflecting disturbances in iron-dependent enzymes such as TPO, affecting overall thyroid hormone metabolism. Bremner et al. [24] found significantly lower serum iron concentrations in participants with subclinical hypothyroidism compared to euthyroid subjects (p=0.001). They also demonstrated a significant association between fT3 and Hb, along with an inverse relationship between TSH, serum iron, and transferrin saturation. Banday et al. [25] in a similar study reported iron deficiency in a significant proportion of patients with primary hypothyroidism. They suggested that thyroid diseases may affect hematopoiesis and thyroid hormone deficiency could lead to bone marrow repression and/or a decrease in erythropoietin production due to reduced O2 requirements. The study also found that thyroid hormones regulate transferrin gene expression. Azizi et al. [26] found a relationship between the frequency of goiter and serum ferritin levels in school children in Iran and reported that the frequency of goiter was related to iron deficiency. In 2015, Refaat [27] reported a higher prevalence of iron deficiency anemia in 52 of 118 non-pregnant females with abnormal thyroid (44%) than in euthyroid pregnant females (14.3%). Subclinical hypothyroidism and iron deficiency anemia are intricately linked conditions that often elude detection until their harmful cycle exacerbates both disorders. Given the apparent manifestation of subclinical hypothyroidism, it is advisable to assess thyroid function in all individuals with iron deficiencies. Similarly, those diagnosed with subclinical hypothyroidism should be evaluated for iron deficiency, and appropriate treatment should be initiated to break the detrimental cycle. In summary, the assessment of the iron profile in patients with subclinical hypothyroidism is crucial as it can influence the treatment strategy and streamline the monitoring of disease progression.

Limitations of the study

While this research offers valuable insights, it's crucial to recognize its limitations. Firstly, the study was conducted at a single center, possibly constraining the broader applicability of the conclusions. Moreover, the sample size was relatively modest, diminishing the statistical power and precision of the outcomes. Additionally, the data collection involved only one-time recordings.

## Conclusions

The prevalence of anemia in subclinical hypothyroidism is significantly elevated, and considering the absence of significant clinical manifestations in the early stages, regular screening through appropriate investigations is advised for early identification, facilitating prompt management. Consequently, our study emphasizes that both overt and subclinical hypothyroidism should be recognized as risk factors for the development of iron deficiency anemia. Adequate treatment is crucial for optimizing the response to therapy in such cases.
